# A Fully Automated Diabetes Prevention Program, Alive-PD: Program Design and Randomized Controlled Trial Protocol

**DOI:** 10.2196/resprot.4046

**Published:** 2015-01-21

**Authors:** Gladys Block, Kristen MJ Azar, Torin J Block, Robert J Romanelli, Heather Carpenter, Donald Hopkins, Latha Palaniappan, Clifford H Block

**Affiliations:** ^1^NutritionQuest, Inc.Berkeley, CAUnited States; ^2^Public Health NutritionSchool of Public HealthUniversity of CaliforniaBerkeley, CAUnited States; ^3^Palo Alto Medical Foundation Research InstitutePalo Alto, CAUnited States

**Keywords:** prediabetes, insulin resistance, diabetes, prevention, obesity, physical activity, internet, world wide web, blood glucose, metabolic syndrome

## Abstract

**Background:**

In the United States, 86 million adults have pre-diabetes. Evidence-based interventions that are both cost effective and widely scalable are needed to prevent diabetes.

**Objective:**

Our goal was to develop a fully automated diabetes prevention program and determine its effectiveness in a randomized controlled trial.

**Methods:**

Subjects with verified pre-diabetes were recruited to participate in a trial of the effectiveness of Alive-PD, a newly developed, 1-year, fully automated behavior change program delivered by email and Web. The program involves weekly tailored goal-setting, team-based and individual challenges, gamification, and other opportunities for interaction. An accompanying mobile phone app supports goal-setting and activity planning. For the trial, participants were randomized by computer algorithm to start the program immediately or after a 6-month delay. The primary outcome measures are change in HbA1c and fasting glucose from baseline to 6 months. The secondary outcome measures are change in HbA1c, glucose, lipids, body mass index (BMI), weight, waist circumference, and blood pressure at 3, 6, 9, and 12 months. Randomization and delivery of the intervention are independent of clinic staff, who are blinded to treatment assignment. Outcomes will be evaluated for the intention-to-treat and per-protocol populations.

**Results:**

A total of 340 subjects with pre-diabetes were randomized to the intervention (n=164) or delayed-entry control group (n=176). Baseline characteristics were as follows: mean age 55 (SD 8.9); mean BMI 31.1 (SD 4.3); male 68.5%; mean fasting glucose 109.9 (SD 8.4) mg/dL; and mean HbA1c 5.6 (SD 0.3)%. Data collection and analysis are in progress. We hypothesize that participants in the intervention group will achieve statistically significant reductions in fasting glucose and HbA1c as compared to the control group at 6 months post baseline.

**Conclusions:**

The randomized trial will provide rigorous evidence regarding the efficacy of this Web- and Internet-based program in reducing or preventing progression of glycemic markers and indirectly in preventing progression to diabetes.

**Trial Registration:**

ClinicalTrials.gov NCT01479062; http://clinicaltrials.gov/show/NCT01479062 (Archived by WebCite at http://www.webcitation.org/6U8ODy1vo).

## Introduction

In the United States, 86 million adults, more than one-third of the population, have pre-diabetes [1]. It has been estimated that over half will eventually progress to type 2 diabetes [2] unless they make changes to their dietary and physical activity behaviors. Therefore, there is an urgent need to reach large numbers of individuals with pre-diabetes with strategies to help them produce long-term behavioral changes and weight management.

Previous interventions intended to prevent diabetes have incorporated various levels and combinations of lifestyle coaching, in-person interaction, and technology-enhanced tools. These various models differ in reach, cost, and effectiveness. Intensive personal and group coaching can produce medically significant changes in those persons with pre-diabetes who choose to participate, as demonstrated by the Diabetes Prevention Program (DPP) [3]. While efficacious, such interventions are costly [4] and challenging to implement [5].

Adaptations of the DPP model in community settings [5-7] that rely on group meetings and frequent in-person contact require substantial organizational skills and professional or semi-professional implementation [8]. Equally important is the reluctance of many, even those quite highly motivated, to dedicate the time to attend a series of onsite meetings. Costs remain substantial [4,8] and effectiveness modest [5,9-11].

Given the well-established transience of weight loss for most people, interventions that can produce robust establishment of new habits and that also permit long-term reinforcement and support in the future are called for. Internet-delivered applications, if proven sufficiently effective, could meet a substantial part of that need. A number of such efforts have been described [9,12-16]. A meta-analysis [9] found that among electronic media-assisted programs, there was a statistically significant but modest mean weight loss of 4.2% body weight. However, often such programs rely on some form of contribution by human counselors in addition to the technological component, which substantially increases cost and will prevent the large economies of scale that a fully automated system would provide.

Fully automated technological approaches have been found to improve diet and/or physical activity behaviors [17,18]. Alive!, which forms the basis of the diabetes prevention program described here, was shown in a randomized trial to produce significant improvements in physical activity and diet [19,20]. However, fully automated or nearly fully automated programs have been studied only rarely for effectiveness on weight loss [12], and almost not at all for diabetes prevention.

To address the need for a diabetes prevention program that can reach thousands or millions of pre-diabetics with an evidence-based intervention, we designed the Alive-PreventDiabetes (Alive-PD) program. Alive-PD is a newly developed, fully automated, 1-year behavior change program to prevent the progression of pre-diabetes to diabetes. Like its precursor program [19,20], it emphasizes lasting lifestyle change in diet and physical activity, which can lead to improvements in blood sugar and weight. It is a stand-alone intervention, although it can also be used to supplement clinician recommendations or to provide long-term support following pre-diabetes classes. This paper describes the Alive-PD program and describes the protocol of the randomized trial currently under way to evaluate its efficacy.

## Methods

### Study Design Overview

The Alive-PD study is a randomized parallel-group controlled trial of the Alive-PD program versus a 6-month-delayed control group among participants with pre-diabetes. The primary outcome measures are change in hemoglobin A1c (HbA1c) and fasting glucose after 6 months. After the study began, the follow-up period was extended to 12 months for both arms. We hypothesize that those in the intervention group will achieve statistically significantly greater reductions in fasting glucose and HbA1c than a delayed-entry control group at 6 months post baseline and that the intervention group will retain a statistically significant change at 1 year. Clinic assessments and laboratory data are collected at 3, 6, 9, and 12 months. The trial is ongoing.

This randomized clinical trial is being conducted in collaboration with the Palo Alto Medical Foundation Research Institute (PAMFRI). The study design and materials were approved by the Institutional Review Boards of NutritionQuest and of the Palo Alto Medical Foundation (PAMF). See Multimedia Appendix 1 for the CONSORT-EHEALTH checklist [21].

### Participant Eligibility Criteria

Participants were recruited from a primary care health center of PAMF, a community-based multi-specialty group practice in northern California. We used the following inclusion criteria for participation: primary care patients with pre-diabetes, age 30-69 years old, BMI ≥27 (BMI >25 for Asian race/ethnic subgroups only), fasting glucose measure in the pre-diabetic range (100-125 mg/dL) or HbA1c in the pre-diabetic range (5.7-6.4%) or physician diagnosis of pre-diabetes, HbA1c ≥5.3% and fasting glucose ≥95 mg/dL (added after study under way), a visit to PAMF primary care department within the past 5 years, access to email and Internet, and able to read and understand English. Those meeting the following exclusion criteria were not eligible: presence of medical conditions contraindicating gradual adoption of moderate physical activity, current use of diabetes medications or weight loss medications, currently pregnant or planning to become pregnant during the period of the study, currently doing more than 150 minutes/week of moderate/vigorous physical activity *and* currently on a low-carbohydrate diet (by self-report), and current participation in another clinical trial.

### Procedures

#### Recruitment and Screening Process

**Figure 1:**
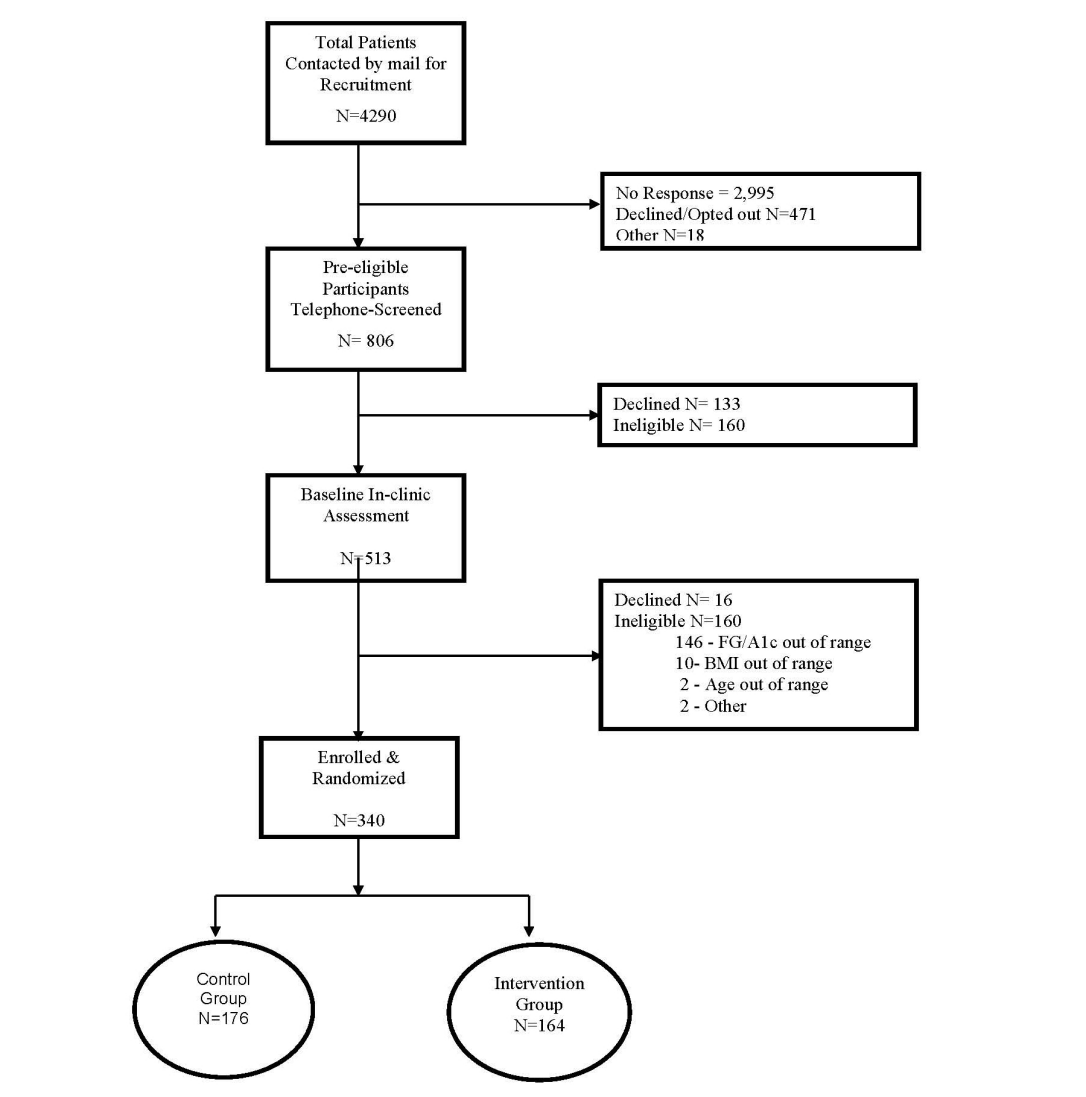
Flowchart of participant screening, recruitment, and randomization.

Potential participants were pre-screened and identified through an electronic health records (EHR) query using broad eligibility criteria of a recent glucose or HbA1c within the pre-diabetes range, in addition to other demographic criteria (Figure 1). In addition to the EHR query, posters were placed in the lobby of the PAMF clinics. Study invitation letters were mailed to potentially eligible participants, with phone follow-up to explain the study, conduct further eligibility assessment, and invite participation. Those who remained eligible and were interested in participating were invited to attend the baseline visit at the PAMF Research Institute for confirmation of eligibility based on point-of-care laboratory testing for glucose and HbA1c. Subjects were enrolled in the study between February and June 2014.

#### Baseline Assessment and Clinic Visit

At the baseline clinic visit, HbA1c, fasting glucose, total and high-density lipoprotein (HDL) cholesterol, triglycerides, and low-density lipoprotein (LDL) by calculation were assessed through point-of-care whole blood samples (Siemens DCA Vantage point-of-care analyzer [22] and Alere Cholestech LDX point-of-care analyzer [23] respectively). In addition to meeting other eligibility criteria, subjects were deemed eligible if either HbA1c and/or fasting glucose fell within the pre-diabetic range, and neither measure reached the diabetic range. A number of subjects who were eligible by one measure were found to have quite low HbA1c or fasting glucose; a further restriction, HbA1c ≥5.3% and glucose ≥95, was added after the study had begun. Prospective participants with one measure in the diabetes range and the other in the pre-diabetes range were deemed provisionally eligible pending physician review and verification of pre-diabetes status. Signed informed consent was obtained.

Other biometric measures included blood pressure, height, weight, and waist circumference, measured according to the procedures recommended by the Airlie Conference. In addition, a baseline questionnaire was administered, assessing medication use, adverse events and participation in activities relevant to weight loss, diet, and physical activity. With separate consent, a blood sample was obtained by FTA (Flinders Technology Associates) card for future research on genetic analysis of the intervention’s efficacy and disease progression.

Once enrolled and confirmed eligible, subjects received brief (5-10 minutes) instruction that they were at risk for developing diabetes and that increased physical activity as well as changes in their dietary behaviors could help prevent progression to diabetes. Research staff assisted participants in signing into an account for the Alive-PD Web-based program, to confirm to the Alive-PD system that they had completed the informed consent and to provide their email address and password to the system. All subsequent communications from and interactions with the electronic Alive-PD program took place outside of the clinic.

#### Subsequent Clinic Visits

At 3, 6, 9, and 12 months, the same biometric and questionnaire measures are repeated. An adverse events questionnaire is also administered at each follow-up.

#### Randomization and Blinding

After the baseline clinic visit and the initial account set-up, randomization and delivery of the intervention is conducted electronically by the Alive-PD program, completely independent of clinic staff. After leaving the clinic, participants are sent an introductory email from the automated Alive-PD system, with further information and questionnaires. These and all further online interactions are done at a time and place of the participants’ choosing. An online baseline questionnaire is required, to provide demographic and BMI information required for randomization and to provide brief eating habits and physical activity data for baseline comparison of intervention and control groups. Participants are randomized by computer algorithm, with blocked stratified randomization to achieve balance on sex, BMI (above/below 35), and race/ethnicity (white non-Hispanic, other). Participants are informed by the system by email about whether they have been randomized to begin the program immediately or after a 6-month delay. Those in the delayed group receive no further contact until a reminder to complete a 3-month and 6-month online follow-up questionnaire. Those randomized to begin immediately receive a more extensive diet and activity questionnaire.

#### Reimbursement

Participants are reimbursed for attending the clinic and providing the biometric outcome data. They receive a US $25 gift card at the 3-month clinic visit and a US $50 gift card at the 6-month clinic visit. If they re-consent to participate in the study extension, they receive a US $25 gift card at the 9-month clinic visit and a US $50 gift card at the 12-month clinic visit.

### Intervention

#### Overview

**Figure 2:**
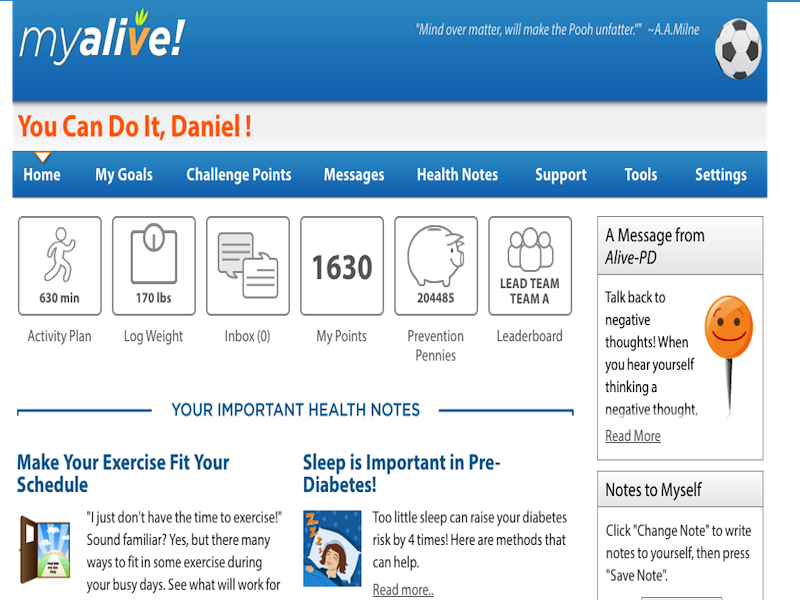
Alive-PD personal home page. Users are taken here when they choose goals in the weekly email. Or they may go here directly at any time -- for example, to log, check or send messages, get automated coaching. Further down the page are infographics and quizzes. If they hadn't yet chosen or reported on goals this week, the home page would show the week's suggested goals to choose, and request a report on last week's goals.

Alive-PD is a fully automated intervention, with no human coaching or advice. It is delivered via an individualized website and interactive emails and is supplemented by a mobile app and automated phone and print modules. Participants complete a diet and physical activity assessment online at baseline, followed by immediate automated individualized feedback. They also complete an activity planning tool to guide improvements in aerobic activity. Participants then engage in a program of weekly tailored goal setting and tracking over the next 6 months and every other week for the subsequent 6 months. Participants work simultaneously on dietary and physical activity behaviors. Targeted behavior changes include increasing physical activity, decreasing added sugars and some starchy carbohydrates, decreasing saturated and trans fats, increasing fruit and vegetable consumption, and achieving modest weight loss or weight maintenance. Participants are invited (but not required) to choose a long-term weight loss target of 5%, 7%, or 10% of body weight. Changes in food type and reduction in portion size are emphasized as a means of reducing calorie intake, rather than specific calorie targets or counting. The focus throughout is on the objective of lowering hemoglobin A1c and preventing diabetes. See Figure 2 and Multimedia Appendix 2 for screenshots and other information.

#### Features of the Alive-PD Program and Website

The program begins with an online baseline questionnaire that assesses the individual’s intake of added sugars, carbohydrates, saturated and trans fats, fruits and vegetables, and time spent in moderate and vigorous intensity activity, walking, sedentary activities and strength training. Questionnaire content is tailored to the participant’s reported ethnic dietary habits. Analysis occurs with each response, allowing for continuous and relevant feedback on the individual’s reported diet and activity, in relation to national guidelines and type 2 diabetes risk factors. The questionnaire responses provide the basis for tailoring specific goals and tips for changes in diet, physical activity, and weight control that the program will recommend to the participant throughout the program. Questions on lifestyle, diet, and physical activity preferences, and psychosocial characteristics form the basis of additional tailoring.

Weekly tailored goal setting is one of the key behavioral features of Alive-PD. In addition to long-term goal setting, individually relevant small-step goals are suggested weekly, developed by an algorithm that draws from the participant’s questionnaire responses, the participant-constructed activity plan, and from an extensive database of potential goals. For example, participants with added sugar intake above the recommended level are offered weekly goals targeting the individual’s own reported sources of added sugars. The goal recommendation algorithm adjusts in response to past performance.

The system suggests to participants that they select one eating habits goal and one physical activity goal from among those suggested each week. However, they may select any number of goals and/or may write their own goals each week. The program also prompts them each week to report on their success at the previous week’s goals. The number and type of successful goals is tracked for the respondent. Mid-week reminders about the goals chosen that week serve to keep the participant engaged and the overall objective salient. Participants who have not responded by mid-week by choosing a goal are reminded by email and/or mobile phone of their self-reported motivations for joining the program and are encouraged again to commit to 1-2 goals for the remainder of the week.

An interactive activity planning tool forms the basis of physical activity goal setting. Alive-PD automatically creates a recommended activity plan based on a participant’s measured activity level, but the participant may edit the plan at any time. The default target is 150 minutes per week, but those already achieving that level may aim higher. As participants interact with each setting, their activity plan is instantly recomputed and drawn onscreen. Their saved plan guides the tailored aerobic activity goals recommended to the participant each week.

Two weekly Health Notes, developed by Certified Diabetes Educators and behavioral experts, provide health information. Topics include subjects such as the role of diet and physical activity in preventing diabetes and other chronic diseases, physical activity guidelines, dietary sources of saturated fat and sugars, portion control, and behavioral strategies including self-monitoring, mindfulness, stress reduction, and sleep. Each Health Note includes a quiz, and participants may rate the Health Note and share it on social media. Delivery of the Health Notes may be tailored to the ethnicity of the participant.

The program promotes social support through a team system, which allows participants to send messages to each other with either a pre-drafted motivational message or a personalized message of support. The system also encourages participants to elicit the support of their personal social network. Participants may invite friends and family to the program, and those who accept receive weekly emails from the system, which include the Health Notes the participant is reading that week and a function to send a message to the participant’s Alive-PD inbox.

Other features include a Daily Tracker to log weight, activity, and dietary intake, with automatic graphing over time; an automated coaching tool to help participants overcome common barriers; weekly infographics that reinforce core messages of the program; downloadable worksheets; and links to external resources.

Program engagement strategies combine intrinsic and extrinsic motivators, with individual and optional team aspects and software gamification features. At the individual level, interaction with central components of the intervention is incentivized using a points system. Participants are awarded points for the following behaviors: setting and reporting goals; successfully accomplishing goals; reading Health Notes and taking the related quizzes; logging body weight, activity, or dietary intake; and sending support messages to other Alive-PD participants. Points can be redeemed for modest monetary rewards or may be contributed to a pooled donation to the American Diabetes Association. At the team level, teams compete for small monetary rewards based on the greatest average HbA1c reduction in each 3-month period.

#### Automated Phone and Print

The content of these Alive-PD modules was developed by nutrition and physical activity professionals, through collaboration with Stanford and Brown University experts. With these technologies, individually tailored print and phone messages are delivered automatically by computer algorithm. They make use of the participant’s baseline responses to factors such as barriers and motivations for behavior change, participant real-time dropout or adherence status, and ongoing participant reports on success at goals undertaken in the Alive-PD program. Individually tailored printed materials are sent monthly. Automated individually tailored phone coaching is delivered every 2 weeks via Interactive Voice Response technology (IVR). Participant responses to the IVR questions are recorded in the system and used to inform subsequent IVR interactions. Both phone and print are entirely automated and require no real-time professional or semiprofessional input.

#### Alive-PD Mobile App

Alive-PD includes an Android and iPhone app that extends some of the program functionality to the participant’s mobile phone. The app allows participants to select Alive-PD weekly goals, report on accomplishment of previous goals, and set calendar reminders. The program also reinforces selected goals via push notifications and coaxes potential dropouts to engage with the program. The mobile phone app was introduced after approximately a quarter of subjects had begun the trial, at which time its availability was announced to all participants.

#### Online Assessment of Non-Physiologic Outcomes

Physiologic outcomes, which are the primary outcomes, are assessed by anthropometric or biometric measures in the clinic, as stated above. Other outcomes are assessed by self-administered questionnaire or through automated capture of use metrics. These include factors such as number of weeks in which a goal was chosen, number of goals chosen, number of goals achieved, number of team support messages sent, and number of quizzes answered. Changes in physical activity and eating habits are assessed by online modified Block questionnaire. Satisfaction with the program is also assessed by online questionnaire.

#### Theoretical Basis

Alive-PD’s intervention strategies are based on principles derived from several models and bodies of behavior change research. The basic objective, derived from learning theory and other habit formation research [24-26] is to have participants gradually incorporate new eating and physical activity behaviors into their daily lives until the behaviors are both habitual and substantial enough to reduce diabetes risk. A focus on small, achievable, personally relevant goals provides continued reward from accomplishing those goals.

Strategies and features of the program are consistent with several other bodies of research, including models centering on cues and triggers [27,28], social cognitive theory [29,30], and the theory of planned behavior [31]. Concepts from behavioral economics [28,32] underlie suggestions for making desired behaviors more convenient and undesired behaviors less convenient. In addition, strategies have been influenced by research on willpower showing the neurophysiological limits of sustained cognitive control for behaviors like dieting [33]. Several empirically validated positive psychology methods [34,35] promote increased resilience and optimism, helpful when undertaking major changes. Techniques based on mindfulness research [36,37] can provide support in the face of cravings. The program’s continuing flow of information on the consequences of diabetes, the ability to reduce one’s risk and skills for doing so is consistent with the health belief model [38]. Promotion of social support through the messaging system, teams, and inclusion of real-world friends and family can provide reinforcement of desirable changes, buffering of stress, and continuing engagement. Further maximizing of engagement through elements such as gamification provides continuing salience of issues around health, nutrition, and physical activity. In sum, techniques consistent with a variety of theories and models provide support for individual differences and for individuals at different times, as they gradually adopt new habits.

### Measures and Data Collection

The primary outcome of Alive-PD is change in HbA1c and fasting glucose at 6 months from baseline, assessed through point-of-care whole blood samples (see Baseline Assessment and Clinic Visit, above). Secondary endpoints are clinic-measured changes in other biometric measures (weight, waist circumference, lipids and other metabolic syndrome factors, and blood pressure). We will examine retention of changes at 12 months in a pre-post analysis. In addition, we will examine self-reported behavior changes in eating and physical activity.

### Statistical Analysis

#### Sample Size

Sample size was estimated using data from Davis et al [39], who conducted a dietary intervention in patients with diabetes. They reported standard deviations for change in HbA1c of 0.9-1.4% for one arm of the study. With an SD of 1.4 and an alpha of .05, a final sample of 268 participants would provide 80% power to detect a minimum detectable difference in change for HbA1c of 0.48. We planned to enroll 314 persons to achieve a completed sample of 268 after a 15% dropout rate.

#### Data Analysis

The primary approach for analyses of treatment effects on glycemic markers (HbA1c and glucose) and other biometric markers will be on the intention-to-treat population. Variables missing at follow-up will be imputed using a maximum likelihood approach. We will also repeat analyses on the per-protocol population, in which effects will be assessed among those providing follow-up biometric data and in relation to the participants’ degree of interaction with the program. Outcomes will be analyzed using linear regression models. Change in the outcome measures (HbA1c, glucose, or weight) will be included in regression models as the dependent variable, and treatment group will be included as the primary predictor variable (intervention vs delayed control). The baseline value of the outcome measure of interest will be included as a covariate. Normality, interaction, and potential confounding will also be investigated, and potential confounders will be included as appropriate. We will also examine the proportion of participants who achieve and retain categories of body weight loss, and the proportion who move from pre-diabetic to normal HbA1c. These binary outcomes will be modeled in logistic regression models with an approach similar to that described for linear regressions. Clinic staff obtaining the biometric measurements are blinded to treatment group and data analysis is conducted on masked data.

## Results

### Enrollment and Baseline Characteristics

A total of 349 participants completed the Informed Consent, and 340 completed the baseline online questionnaire and were randomized to Alive-PD or 6-month delayed control. The 9 participants who did not complete the baseline questionnaire did not differ in age, sex, race, BMI, or lipids, but did have a significantly lower fasting glucose but higher HbA1c (data not shown).

Baseline characteristics are shown in Table 1. The large proportion of males (68.5%, 233/340) is notable, and quite the opposite of what is commonly seen in health studies. It is also notable that only 45% of the sample were in the pre-diabetic range by HbA1c and that the mean HbA1c is in the normal range.

**Table 1:** Baseline demographics and clinical characteristics.

Variable	Category	All (n=340)	Control (n=176)	Intervention (n=164)	*P* ^a^
Age, years, mean (SD)		55.0 (8.9)	54.8 (9.1)	55.1 (8.8)	.77
Female, n (%)		107 (31.5)	54 (30.7)	53 (32.2)	.75
**Race/ethnicity, n (%)** ^b^					.38
	White	204 (60.0)	104 (59.1)	100 (61.0)	
	Black	7 (2.1)	1 (0.6)	6 (3.7)	
	Asian	63 (18.5)	36 (20.4)	27 (16.5)	
	Hispanic	16 (4.7)	9 (5.1)	7 (4.3)	
	Other	8 (2.3)	5 (2.8)	3 (1.8)	
	Not reported	42 (12.4)	21 (11.9)	21 (12.8)	
**Blood pressure, mmHg, mean (SD)**					
	Systolic	130.4 (14.7)	130.4 (14.5)	130.4 (15.0)	.99
	Diastolic	82.3 (8.4)	82.6 (8.6)	82.0 (8.1)	.45
Weight, lbs, mean (SD)		204.8 (34.8)	205.6 (36.5)	203.9 (32.9)	.64
BMI, kg/m^2^, mean (SD)		31.1 (4.3)	31.2 (4.3)	31.1 (4.4)	.86
Waist circumference, cm, mean (SD)		102.8 (10.8)	103.1 (11.2)	102.5 (10.4)	.59
Glucose, mg/dL, mean (SD)		109.9 (8.4)	109.0 (8.3)	110.2 (8.6)	.52
HbA1c, %, mean (SD)		5.6 (0.3)	5.6 (0.3)	5.6 (0.3)	.94
Total cholesterol, mg/dL, mean (SD)		191.5 (31.7)	191.9 (33.0)	191.2 (30.2)	.83
LDL cholesterol, mg/dL^c^, mean (SD)		116.0 (27.6)	116.5 (27.9)	115.5 (27.3)	.72
HDL cholesterol, mg/dL^c^, mean (SD)		47.3 (14.0)	46.5 (13.0)	47.8 (14.5)	.40
Triglycerides, mg/dL^c^, mean (SD)		144.6 (73.7)	148.2 (68.8)	139.4 (67.3)	.24

^a^Significance of difference between intervention and control.

^b^Native American/Alaskan, Native Hawaiian/Pacific Islander, reported as multiple races or “other”. Ethnicity from EHR.

^c^For LDL: Control, n=172, Intervention, n=161; for HDL: Control, n=176, Intervention, n=162; for triglycerides: Control, n=174, Intervention, n=162; for waist circumference: Control, n=175, Intervention, n=163.

### Preliminary Outcome Results

Preliminary data on treatment-control differences indicate a statistically significant treatment effect on HbA1c, fasting glucose, and body weight (data reported elsewhere). These significant treatment effects were seen despite the fact that the control group achieved a statistically significant (*P*<.01) reduction in HbA1c and weight.

### Preliminary Participation, Retention Results

Dropout rate (defined as permanently declining to attend further clinic visits) as of the 3-month clinic visit is 5.9% (20/340). Also as of the 3-month time point, 78% of the intervention group were still participating actively in the online program, by choosing or reporting on a goal, logging weight or activity, answering a quiz, or sending a message to team members via the online messaging system.

## Discussion

### Summary

The Alive-PD program is one of very few interventions designed as a fully automated algorithm-driven program for persons at risk of developing diabetes. If proven effective in the randomized trial described here, it could reach many of the 86 million US pre-diabetics with an evidence-based behavior-change intervention. In addition, the delivery of the program is highly configurable and can be readily adapted for other medical conditions or for populations with other eating and activity behaviors. Alive-PD will be commercially available.

### Limitations

We will not have the duration or sample size to have a diabetes incidence endpoint. However, we do have the power to assess efficacy for glycemic control and weight loss. Furthermore, the fact that the study is restricted to participants with email and Internet access is a limitation. However, as of August 2011, 92% of American adults used email and the Internet, including 93% of whites, 87% of African Americans, and 88% of Hispanics, and over 50% had a mobile phone. Finally, we recognize that wholly automated interactions, without person-to-person contact, can be limiting for many. However, such a system can reach large numbers at a much lower cost than systems requiring interaction with human coaches. Even modest changes on a proportion of the very large target population could result in major benefits in cost savings and disease prevention. If only 5% of the 86 million Americans with pre-diabetes were reached with an effective automated intervention program, and as few as one-third of them succeeded in maintaining or reducing their pre-diabetic status, that would mean more than 1 million Americans with reduced risk of type 2 diabetes and billions of dollars saved in health care costs.

### Conclusions

Alive-PD is a multi-channel automated intervention to prevent diabetes by lowering or preventing the increase in HbA1c and glucose. If proven effective, Alive-PD would demonstrate how a preventative intervention can be provided with wide reach, high fidelity, and low cost for persons with pre-diabetes.

## Multimedia Appendix 1

CONSORT-EHEALTH checklist V1.6.1 [21].

## Multimedia Appendix 2

Screenshots, Medicine 2.0 2014 talk, Gladys Block.

## Multimedia Appendix 3

NIH reviewers' comments.

## Abbreviations

EHR: electronic health records
HDL: high-density lipoprotein
IVR: Interactive Voice Response
LDL: low-density lipoprotein
PAMF: Palo Alto Medical Foundation
PAMFRI: Palo Alto Medical Foundation Research Institute
RCT: randomized controlled trial
