# Supplementary material for: A Fully Automated Diabetes Prevention Program, Alive-PD: Program Design and Randomized Controlled Trial Protocol
Source: JMIR Res Protoc. 2015 Jan 21;4(1):e3. doi: 10.2196/resprot.4046 (PMC4319077; doi:10.2196/resprot.4046)
Supplement: Supplementary file 3 [file resprot_v4i1e3_app3.PDF]

SUMMARY STATEMENT
(Privileged Communication)

PROGRAM CONTACT:
Paul Cotton
301-402-6423
cottonp@mail.nih.gov

Release Date: **07/01/2010**

Application Number: **1 R44 NR012617-01**

Principal Investigator

BLOCK, GLADYS PHD

Applicant Organization: BERKELEY ANALYTICS, INC.

Review Group: **ZRG1 RPHB-C (10)**
Center for Scientific Review Special Emphasis Panel
Small Business: Risk Prevention and Health Behavior

Meeting Date: **06/23/2010**
Council: **OCT 2010**
Requested Start: **12/01/2010**

RFA/PA: **PA08-114**
PCC: **BSBPC**
Dual PCC: **DKN DUAL**
Dual IC(s): **DK**

Project Title: **Multi-Channel Automated Diet and Physical Activity Intervention for Pre-Diabetics**

SRG Action: **Impact/Priority Score: 29**
Human Subjects: **30-Human subjects involved - Certified, no SRG concerns**
Animal Subjects: **10-No live vertebrate animals involved for competing appl.**
Gender: **1A-Both genders, scientifically acceptable**
Minority: **1A-Minorities and non-minorities, scientifically acceptable**
Children: **3A-No children included, scientifically acceptable**
Clinical Research - not NIH-defined Phase III Trial

Project Year	Direct Costs Requested	Estimated Total Cost
1	198,541	353,731
2	315,577	562,249
3	298,154	531,207
4	302,940	539,734
TOTAL	1,115,212	1,986,921

ADMINISTRATIVE BUDGET NOTE: The budget shown is the requested budget and has not been adjusted to reflect any recommendations made by reviewers. If an award is planned, the costs will be calculated by Institute grants management staff based on the recommendations outlined below in the COMMITTEE BUDGET RECOMMENDATIONS section.

ADMINISTRATIVE NOTE

ADMINISTRATIVE NOTE

RESUME AND SUMMARY OF DISCUSSION: The overall goal of this fast-track application is to adapt an existing email- and net-based wellness program to a product aimed at changing physical activity, diet and weight loss behaviors of pre-diabetics. Some Committee member felt that it has a high potential for success and is soundly based on prior work. Others voiced concern that the base program was a very intense in-person intervention while this is a light touch. In its support, reviewers believed that although this program will not really stand alone, it can be disseminated to a broad population and may have some impact in the context as other supports. The program is likely to be attractive to third party branding. An additional concern related to the conduct of the focus groups. Overall, this balance of reviewer noted strengths and weaknesses resulted in an assessment of the application as being excellent with some minor limitations.

DESCRIPTION (provided by applicant): Significance: Almost 30% of U.S. adults have pre-diabetes. Without weight loss and changes in physical activity and diet, more than 50% of them will eventually develop type 2 diabetes (T2DM), with its increased risk of cardiovascular disease and many other complications. The Diabetes Prevention Program (DPP) proved that lifestyle changes involving weight loss, increased physical activity and dietary changes can delay or prevent the onset of T2DM in pre-diabetics. However, intensive interventions like DPP are expensive (DPP: $1,399/person) and demanding of both patient and professional time. As a result, despite the critical need to prevent progression to T2DM, prevention programs have not been embodied in effective, low-cost, real-world interventions that can assist millions of pre-diabetics. Objectives: The overall goal of this fast-track proposal is to produce a commercializable product aimed at changing physical activity, diet and weight loss behaviors of pre-diabetics. The specific aims are to 1) Adapt an existing successful email/Internet wellness program to focus on prediabetics; 2) Conduct a randomized trial of effectiveness in changing weight, glucose and physical activity in pre-diabetics; 3) Provide data on cost and participation, for commercialization and future phase 3 trials. Methods: "Alive" is an email-delivered intervention to improve physical activity and diet, developed through an R01 to Kaiser Permanente. In a randomized controlled trial, Alive significantly improved all of its target behaviors. The latest version, through an SBIR grant, has added automated tailored print and phone counseling, social networking and a substantial emphasis on weight control. Alive will now be modified to focus on pre-diabetes and the behaviors required for its effective control. The modified program will be delivered over I year through 3 channels (email/internet, phone, print), and will use computerized algorithms to deliver highly individualized weekly goal-setting and motivational support. In the clinical trial, pre-diabetics will be identified through the Kaiser Permanente member database. At clinic visits at baseline, 3 and 12 months, weight and measures of glycemic control will be obtained. Subjects will be randomly assigned to the modified program, Alive-PD, or a control condition representing usual care of pre-diabetics. There is statistical power to detect a clinically meaningful difference between the two groups in weight loss and glycemic control (A1c). Effectiveness, proportion reached, participation and cost will be reported. Impact: A low-cost program such as this could reach thousands or millions of prediabetics with a behavior- change intervention, at a fraction of the cost of in-person or group approaches. If proven effective at improving weight loss and glycemic control in a randomized trial, it could become an important component of the standard of care for prediabetics as a stand-alone service, as a complement to pre-diabetes education classes, and as an important reinforcer of physician and nurse practitioner recommendations.

PUBLIC HEALTH RELEVANCE: Pre-diabetes affects almost 30% of U.S. adults, many of whom will progress to type 2 diabetes unless they lose weight and increase their physical activity. Currently, effective interventions are expensive and burdensome. This project will modify and test an effective

program that could be widely used to help pre-diabetics lose weight and be more active and prevent the progression to diabetes.

CRITIQUE 1:

Significance: 1
Investigator(s): 3
Innovation: 3
Approach: 2
Environment: 1

Overall Impact:

Strengths

- The application targets an important and increasing health issue in the US, the growth of type 2 diabetes. The application will attempt to address this problem by designing and testing an email/internet based lifestyle modification program for individuals who have prediabetes. The program will assist these people to prevent diabetes or delay type 2 diabetes.
- An effective and economic program targeting prediabetic individuals is an innovation. If the investigators can have a beneficial impact on the health behavior of this group, it could have a significant impact for patients, healthcare organizations, and payers.
- This is an exceptional group of experienced investigators and advisors who have the necessary resources and collaborations to complete this project.
- The application will modify a proven and successful product to address prediabetes.

Weaknesses

- While a strong team of investigators, there is no behavioral scientist who specializes in diabetes. It is also curious that there is no one associated with the Diabetes Prevention Program is on the advisory board since the DPP is a crucial resource for the application.
- Dr. Gladys Block is the Principal Investigator on two existing ongoing grants, does she have the time to direct a third?

1. Significance:

Strengths

- This prevention of prediabetes application addresses a critical health problem and has great commercial potential. If successful, the potential market would be patients, providers, healthcare providers, and payers.

Weaknesses

- None.

2. Investigator(s):

Strengths

- This team of investigators, advisors, and collaborators has the experience and skill to complete the activities outlined. This is a distinguished group of researchers and clinicians.

Weaknesses

- One missing component of investigator team is an experienced diabetes behavioral scientist. The San Francisco bay area has several distinguished individuals in this field.
- Similarly, the application indicates substantial reliance on the Diabetes Prevention Program for Alive-PD modifications. It would have strengthened the application if a member of the advisory board had been an investigator of that study.
- Dr. Gladys Block is already the Principal Investigator on two existing ongoing grants. This may limit her availability to direct this project.

3. Innovation:

Strengths

- The innovative aspect of this application is the development of an effective and economical prevention program targeting individual with prediabetes. If successful, this product has a substantial market.

Weaknesses

- As the investigators state, an internet-based program is not innovative. Prevention programs targeting prediabetic individuals are available.

4. Approach:

Strengths

- All of the proposed activities are ethologically sound and appropriate.
- The pilot in Phase I and the randomized trial in Phase II strengthen the project.

Weaknesses

- On page 120 under the Feasibility Criteria, there should be a “decrease or improvement in glucose” not just a “change in glucose.”
- The investigators state that this application has no overlap with their existing grant “Diet and Activity Promotion Among Older Working Adults.” Given the application deals with prediabetes, this reviewer is not certain how these two could not overlap.

5. Environment1:

Strengths

- The investigators have the resources, the support, and the collaborations for the proposed activities.

Weaknesses

- None.

Fast Track (Type 1 R42 and Type 1 R44 applications):

Acceptable

- The application's Phase I has clear and measureable milestones. The investigators have submitted a Commercialization Plan.

Protections for Human Subjects:

Acceptable Risks and Adequate Protections

- One item of concern is the investigators comment that in the Phase I focus groups "Topics will be non-sensitive." Why do they think discussing diabetes or prediabetes is "non-sensitive"? It will be sensitive to these individuals and the investigators need to be aware of this.

Data and Safety Monitoring Plan (Applicable for Clinical Trials Only):

Acceptable

- o Given the project's intervention and the investigators' plan, a Data and Safety Monitoring Board is not necessary. The plan provides adequate protection for participants.

Inclusion of Women, Minorities and Children:

G1A - Both Genders, Acceptable

M1A - Minority and Non-minority, Acceptable

C3A - No Children Included, Acceptable

- The application focuses on the prevention of type 2 diabetes. This is primarily an adult disease.

Vertebrate Animals: Not Applicable (No Vertebrate Animals)

Biohazards: Not Applicable (No Biohazards)

Budget and Period of Support:

Recommended budget modifications or possible overlap identified:

- Could be possible overlap with their existing grant "Diet and Activity Promotion Among Older Working Adults". If determined not to be overlap, then budget is acceptable.

Additional Comments to Applicant:

- Unsure why certain sections (e.g., the population characteristics and sources of materials) were placed under Protection of Human Subjects, unless it was to circumvent new page limits. Such information is better placed in the Research Strategy.

CRITIQUE 2:

Significance: 1
Investigator(s): 1
Innovation: 3
Approach: 2
Environment: 1

Overall Impact:

Strengths

- This project focuses on an extremely important public health problem, pre-diabetes, that affects a significant portion of the population.
- The project focuses on prevention of diabetes which will further prevent increased risk of a variety of other chronic disease outcomes.

Weaknesses

- No major weaknesses noted.

1. Significance:

Strengths

- Addresses and important and underserved public health problem
- Provides a low-cost intervention for a problem (pre-diabetes) for which relatively few resources are available and for which insurance coverage is often lacking.

Weaknesses

- The clinical trial (and materials) will be limited to relatively high SES, English-speaking individuals. Therefore, the overall impact may be somewhat minimized.

2. Investigator(s):

Strengths

- The investigative team is quite strong with the researchers having representation by a variety of scientific disciplines (i.e., behavioral psychology, nutritional epidemiology, statistics, endocrinology, etc.)
- The combination of strong scientific expertise with strong capabilities for implementation is particularly strong.
- The scientific leaders have vast experience with studies of nutrition research and experience with developing products for obesity control, diabetes and nutrition.

Weaknesses

- The scientific team is quite large (although does not appear to be cumbersome) even for a clinical trial of this magnitude.

3. Innovation:

Strengths

- Focusing on pre-diabetics in a minimal cost manner (i.e., via mail and internet) is extremely important because there are a large number of these patients, they are at increased risk of diabetes (and the associated costs) and the subsequent chronic disease sequelae (and their increased costs), but not all pre-diabetics will progress to these more costly conditions.
- Clinicians lack evidence-based programs to reduce the risk that pre-diabetics will develop diabetes.
- Individually tailored messages

Weaknesses

- Web-based provision of such material is not particularly innovative.

4. Approach:

Strengths

- The program is adapting an existing behavior change model and appropriately testing it through a RCT.
- The existing ALIVE! Program has been proven to be effective in changing behavior in a workplace-based context.
- Builds on behavior change theory and scientifically documented evidence suggesting that behavior change can delay and/or prevent development of diabetes in pre-diabetics.

Weaknesses

- The pilot study is incorporated as part of the project. While piloting methods, etc. is critical to the success of an RCT, there are concerns about the conduct of the RCT in the remote chance that the pilot study fails.
- It would be helpful to have some evidence or discussion that the ALIVE! Program has appropriate modules for the pre-diabetic target age (45-74) as compared to the workplace based modules which would target a different age range. Further as of 2008, approximately half of individuals age 65-75 were “on-line”. Some further discussion of the impact of this discrepancy would have been helpful.
- Power does not seem to account for potential losses to follow-up.
- Application suggests that recruitment will continue into early year 3. However, this will not allow for time to follow these last enrollees for 1 year.
- The human protection section indicates that individuals with extremely high blood glucose and blood pressures will be excluded from the study and referred for treatment. However, these are not documented as exclusionary criteria nor are they fully defined.

5. Environment:

Strengths

- Extremely strong environment for conducting this research.
- Berkeley Analytics and Kaiser Permanente have a long track record of conducting this kind of research and have the facilities and technology to make this clinical trial feasible.

Weaknesses

- No major weaknesses noted.

Fast Track (Type 1 R42 and Type 1 R44 applications):

Acceptable

- This application will incorporate both Phase I and Phase II activities. This is likely justified given that the methods are well proven and that little revision is likely required. However, there is a minor concern that in the unlikely event that piloting and early work shows that the project/product will not be successful the methods will need to be revised.

Protections for Human Subjects:

Acceptable Risks and Adequate Protections

- The project has an adequate plan for protection of human subjects.

Data and Safety Monitoring Plan (Applicable for Clinical Trials Only):

Acceptable

- o The investigators will serve to monitor data safety. Given the limited risk associated with this project, this is likely adequate and no DSMB is required.

Inclusion of Women, Minorities and Children:

G1A - Both Genders, Acceptable

M1A - Minority and Non-minority, Acceptable

C3A - No Children Included, Acceptable

- The project will include both genders and all individuals regardless of minority status. Given the study's location, both minority and non-minority subjects will be included. The investigators have appropriately chosen to exclude children as this is a different population with different issues and concerns. All material will be presented in English only and will be presented via internet. This may limit the inclusion of some segments of the population.

Vertebrate Animals: Not Applicable (No Vertebrate Animals)

Biohazards: Not Applicable (No Biohazards)

Budget and Period of Support:

Recommend as Requested

CRITIQUE 3:

Significance: 3
Investigator(s): 3
Innovation: 3
Approach: 3
Environment: 1

Overall Impact:

Strengths

- Almost one-third of U.S. adults have pre-diabetes; more than 50% will eventually develop diabetes.
- Builds on a theory-based intervention that was developed in a federally funded clinical trial ("Alive") and adds components of the DPP.

- Investigators provide a thorough review of internet weight loss programs, as well as provide a detailed justification for why the proposed intervention is needed.
- Social networking and individually-tailored print and phone messages enhance the internet component
- Measures cost and medical care expenditures for each subject in intervention and control to evaluate the cost-effectiveness.
- Linking the intervention with primary care providers improves the potential for commercial distribution.
- The research design is strong and the measures selected are appropriate.
- Experienced team of investigators

Weaknesses

- More information should be provided on the use of the program in the original study.
- More detail regarding how data from the formative evaluation will be analyzed and used to inform the web-based intervention program.
- Need a more detailed process evaluation to understand issues associated with feasibility, acceptability, and adherence.
- Although this program targets the individual and family level, it is important to incorporate information regarding how to address obesity-promoting factors in the home environment and neighborhood environment vs. psychosocial factors alone.

1. Significance:

Strengths

- Fifty-seven million U.S. adults have pre-diabetes, defined as either impaired fasting glucose or impaired glucose tolerance (1). Over half of pre-diabetics will eventually progress to type 2 diabetes (T2DM) (2) unless they reduce their weight and increase their physical activity. Risk of cardiovascular disease also increases linearly with increasing glucose, even at levels below the definition of diabetes (3). The annual cost of diabetes in the U.S. is $174 billion.
- Behavioral programs that can be widely distributed are desperately needed.
- Although many researchers have advocated for environmental approaches to address obesity and its related risk factors, individual interventions are an important aspect of obesity prevention and treatment.

Weaknesses

- No major weaknesses noted.

2. Investigator(s):

Strengths

- Strong team of investigators.
- Dr. Block has extensive experience in this area and has successfully completed similar SBIR grants. As well as an established program in dietary assessment.

Weaknesses

- No major weaknesses noted.

3. Innovation:

Strengths

- No major strengths noted.

Weaknesses

- The program itself is not that innovative. Builds on existing evidence from previous studies and attempts to disseminate it to a broader population.

4. Approach:

Strengths

- Includes a both a short-term feasibility trial and larger trial.
- Measures cost and medical care expenditures for each subject in intervention and control to evaluate the cost-effectiveness.
- Little information is provided regarding how the original intervention was received by participants. How much were the Alive materials used by participants in the original study? Did they read the emails and print materials?

Weaknesses

- Need more detail about how the focus groups and qualitative feedback from the formative assessment will be analyzed and incorporated into intervention development and refinement.
- More detail on the process evaluation.
- Although this program targets the individual and family level, it is important to incorporate information regarding how to address obesity-promoting factors in the home and neighborhood environment vs. psychosocial factors alone. Print materials should include information about local resources.

5. Environment:

Strengths

- The environment is adequate to support the proposed project.

Weaknesses

- No major weaknesses noted.

Fast Track (Type 1 R42 and Type 1 R44 applications):

Acceptable

Protections for Human Subjects:

Acceptable Risks and Adequate Protections

Data and Safety Monitoring Plan (Applicable for Clinical Trials Only):

Acceptable

Inclusion of Women, Minorities and Children:

G1A - Both Genders, Acceptable

M1A - Minority and Non-minority, Acceptable

C3A - No Children Included, Acceptable

Vertebrate Animals: Not Applicable (No Vertebrate Animals)

Biohazards: Not Applicable (No Biohazards)

Budget and Period of Support:

Recommend as Requested

- As indicated by the investigator the budget is high.

THE FOLLOWING RESUME SECTIONS WERE PREPARED BY THE SCIENTIFIC REVIEW OFFICER TO SUMMARIZE THE OUTCOME OF DISCUSSIONS OF THE REVIEW COMMITTEE ON THE FOLLOWING ISSUES:

PROTECTION OF HUMAN SUBJECTS (Resume): ACCEPTABLE
No concerns.

INCLUSION OF WOMEN PLAN (Resume): ACCEPTABLE
The project will include both genders and all individuals regardless of minority status.
INCLUSION OF MINORITIES PLAN (Resume): ACCEPTABLE
The project will include both genders and all individuals regardless of minority status.

INCLUSION OF CHILDREN PLAN (Resume): ACCEPTABLE
Children are appropriate excluded.

COMMITTEE BUDGET RECOMMENDATIONS: The budget was recommended as requested.

ADMINISTRATIVE NOTE:
During the review of this application, reviewers noted that page limits for one or more sections of the application may have been circumvented by including excess text in one or more application sections that do not have specified page limits (e.g., Protection of Human Subjects).

MEETING ROSTER

Center for Scientific Review Special Emphasis Panel
CENTER FOR SCIENTIFIC REVIEW
Small Business: Risk Prevention and Health Behavior
ZRG1 RPHB-C (10) B
June 23, 2010 - June 25, 2010

CHAIRPERSON

DI NOIA, JENNIFER , PHD
RESEARCH SCIENTIST
DEPARTMENT OF SOCIOLOGY
WILLIAM PATERSON UNIVERSITY
WAYNE, NJ 07470

MEMBERS

ASCIONE, FRANK J, DP, PHD
DEAN AND PROFESSOR
COLLEGE OF PHARMACY
UNIVERSITY OF MICHIGAN
ANN ARBOR, MI 481091065

BOCK, BETH C, PHD
ASSOCIATE PROFESSOR
CENTER FOR BEHAVIORAL AND PREVENTIVE MEDICINE
MIRIAM HOSPITAL
PROVIDENCE, RI 02903

BRAVEMAN, BRENT , PHD
DIRECTOR OF PROFESSIONAL EDUCATION/CLINICAL PROFESSOR
DEPARTMEN T OF OCCUPATIONAL THERAPY
UNIVERSITY OF ILLINOIS AT CHICAGO
CHICAGO, IL 60612

CHASTAIN, GERALD WOODYARD
VICE PRESIDENT
DEPARTMENT OF DEVELOPMENT,
TECHNOLOGY AND MEDIA
LEARNING MULTI-SYSTEMS, INCORPORATED
MADISON, WI 53713

DODGE, KAREN CHRISTINE, PHD
DIRECTOR OF RESEARCH
HANLEY CENTER, INC
MILLER SCHOOL OF MEDICINE
UNIVERSITY OF MIAMI
WEST PALM BEACH, FL 33407

DREWS-BOTSCH, CAROLYN , PHD
ASSOCIATE PROFESSOR OF EPIDEMIOLOGY
DEPARTMENT OF EPIDEMIOLOGY
ROLLINS SCHOOL OF PUBLIC HEALTH
AND JAMES T. LANEY SCHOOL OF GRADUATE STUDIES
EMORY UNIVERSITY
ATLANTA, GA 30322

DUHAMEL, KATHERINE N, PHD
ASSOCIATE ATTENDING PSYCHOLOGIST
DEPARTMENT OF PSYCHIATRY
AND BEHAVIORAL SCIENCES
MEMORIAL SLOAN-KETTERING CANCER CENTER
NEW YORK, NY 10022

FETTEROLF, DONALD E., MD
PRINCIPAL
FETTEROLF HEALTHCARE CONSULTING
PITSBURGH, PA 15222

FITZGERALD, JAMES THOMAS, PHD
ASSOCIATE PROFESSOR
DEPARTMENT OF MEDICAL EDUCATION
UNIVERSITY OF MICHIGAN
ANN ARBOR, MI 481090201

HARNETT, BRETT M MS IS
ASSISTANT PROFESSOR
DEPARTMENT OF SURGERY
UNIVERSITY OF CINCINNATI
CINCINNATI, OH 45267

HOLLAND, JOHN K
CEO AND DIRECTOR OF RESEARCH
LIFELINK HOME LLC
LAKE KATRINE, NY 12449

ISASI, CARMEN R, MD, PHD
ASSISTANT PROFESSOR
DEPARTMENT OF EPIDEMIOLOGY AND
POPULATION HEALTH
ALBERT EINSTEIN COLLEGE OF MEDICINE
YESHIVA UNIVERSITY
BRONX, NY 10461

LANCASTER, BLAKE M, PHD
ASSISTANT PROFESSOR
DEPARTMENT OF PSYCHOLOGY
MUNROE-MEYER INSTITUTE AT THE
UNIVERSITY OF NEBRASKA MEDICAL CENTER
OMAHA, NE 68106

MITCHELL, ROGER E., PHD
ASSISTANT PROFESSOR
DEPARTMENT OF PSYCHOLOGY
NORTH CAROLINA STATE UNIVERSITY
RALEIGH, NC 27695

NOYES, CHARLOTTE
PRINCIPAL
NOYES RESEARCH CONSULTING
CARMEL, CA 93923

ODOMS-YOUNG, ANGELA M, PHD
ASSISTANT PROFESSOR
DEPARTMENT OF KINESIOLOGY AND NUTRITION
UNIVERSITY OF ILLINOIS AT CHICAGO
CHICAGO, IL 60612

OOI, WEE LOCK, DRPH
SENIOR RESEARCHER
MASSACHUSETTS DEPARTMENT OF PUBLIC HEALTH
OFFICE OF STATISTICS AND EVALUATION
BUREAU OF FAMILY AND COMMUNITY HEALTH
NEWTON, MA 02108

OSBORN, CYNTHIA J, PHD
PROFESSOR
COUNSELING AND HUMAN DEVELOPMENT
SERVICES PROGRAM
KENT STATE UNIVERSITY
KENT, OH 44242

PAINE, LISA L., DRPH
PRINCIPAL AND SENIOR CONSULTANT
THE HUTCHINSON DYER GROUP
CAMBRIDGE, MA 02238

PHELAN, KIERAN J, MD
ASSOCIATE PROFESSOR OF PEDIATRICS
DEPARTMENT OF PEDIATRICS
DIVISION OF HEALTH POLICY AND CLINICAL
EFFECTIVENESS, CHILDREN'S HOSPITAL MEDICAL
CENTER
UNIVERSITY OF CINCINNATI
CINCINNATI, OH 452293039

RESNICK, HELAINE E., PHD
DIRECTOR OF RESEARCH
INSTITUTE FOR THE FUTURE OF AGING SERVICES
AMERICAN ASSOCIATION OF HOMES
AND SERVICES FOR THE AGING
WASHINGTON, DC 20008

ROGERS, MARY , PHD
ASSOCIATE DIRECTOR, RESEARCH SCIENTIST
SANFORD HEALTH RESEARCH
HEALTH DISPARITIES RESEARCH
SPEARFISH, SD 57783

SHEGOG, ROSS , PHD
ASSISTANT PROFESSOR
CENTER FOR HEALTH PROMOTION
AND PREVENTION RESEARCH
SCHOOL OF PUBLIC HEALTH
UNIVERSITY OF TEXAS
HOUSTON, TX 77030

SIKORSKII, ALLA , PHD
ASSISTANT PROFESSOR
DEPARTMENT OF STATISTICS AND PROBABILITY
MICHIGAN STATE UNIVERSITY
EAST LANSING, MI 48824

SPRENGELMEYER, PETER G, PHD
EXECUTIVE DIRECTOR
OREGON SOCIAL LEARNING CENTER
COMMUNITY PROGRAMS
EUGENE, OR 97401

SWARTZ, ANN M, PHD
ASSOCIATE PROFESSOR
DEPARTMENT OF HUMAN MOVEMENT SCIENCES
UNIVERSITY OF WISCONSIN-MILWAUKEE
MILWAUKEE, WI 532010413

THOMBS, DENNIS L, PHD
ASSOCIATE PROFESSOR
DEPARTMENT OF BEHAVIORAL SCIENCE AND
COMMUNITY HEALTH
COLLEGE OF PUBLIC HEALTH AND HEALTH
PROFESSIONS
UNIVERSITY OF FLORIDA
GAINESVILLE, FL 32610

THOMPSON, DEBORAH I, PHD
USDA/ARS SCIENTIST/NUTRITIONIST
DEPARTMENT OF PEDIATRICS
CHILDREN'S NUTRITION RESEARCH CENTER
BAYLOR COLLEGE OF MEDICINE
HOUSTON, TX 77030

THOMPSON, DIXIE L, PHD
PROFESSOR AND DEPARTMENT HEAD
DEPARTMENT OF EXERCISE, SPORT
AND LEISURE STUDIES
THE UNIVERSITY OF TENNESSEE
KNOXVILLE, TN 37996

WARD, SANDRA E, PHD
PROFESSOR
CENTER FOR PATIENT-CENTERED INTERVENTIONS
SCHOOL OF NURSING
UNIVERSITY OF WISCONSIN-MADISON
MADISON, WI 53792

SCIENTIFIC REVIEW ADMINISTRATOR

GUTKIN, CLAIRE E., MPH, PHD
SCIENTIFIC REVIEW OFFICER
CENTER FOR SCIENTIFIC REVIEW
NATIONAL INSTITUTES OF HEALTH
BETHESDA, MD 20892

GRANTS TECHNICAL ASSISTANT

ROTTER, LEE
EXTRAMURAL SUPPORT ASSISTANT
CENTER FOR SCIENTIFIC REVIEW
NATIONAL INSTITUTES OF HEALTH
BETHESDA, MD 20892

Consultants are required to absent themselves from the room during the review of any application if their presence would constitute or appear to constitute a conflict of interest.
